# Perspective on the ethics of AI at the intersection of nutrition and behaviour change

**DOI:** 10.3389/fragi.2025.1423759

**Published:** 2025-05-09

**Authors:** Mariette Abrahams, Maria Raimundo

**Affiliations:** ^1^ Qina Ltd., Olhao, Portugal; ^2^ Maria Raimundo Consulting, Lisbon, Portugal

**Keywords:** personalized nutrition, behaviour change, artificial intelligence, digital health, longevity

## Abstract

Artificial intelligence (AI) has emerged as a powerful tool, that has the potential to impact society on multiple levels. Increased adoption as well as employment of AI in new product development and business processes have led to heightened interest and optimism on one hand, whilst increasing fears of potential negative societal consequences on the other. The ethics of AI has subsequently become a topical issue for academics, industry players, health practitioners and regulators, who have a goal and responsibility to protect the public and limit widening inequality. Despite the publication of numerous AI ethical frameworks, guidelines and regulations, none have specifically focused on nutrition and behaviour change. Advances in technology, including AI and machine learning, have opened up novel ways to deliver personalization to guide individuals towards healthier behaviours or to manage their conditions. This perspective synthesizes the key topics that intersect in nutrition and behaviour change where AI is leveraged to provide personalized advice. We propose a 7-pillar framework to guide the development of ethical and transparent AI solutions to build consumer and practitioner trust.

## Introduction

Personalized nutrition is defined as a service or product that uses individual-specific information, is based on evidence-based science, and aims to empower consumers to make positive, sustainable dietary changes for health improvement, maintenance, or disease-specific benefits (Clabbers et al., 2021). The personalized nutrition market is currently valued at around $16 billion (MarketsandMarkets, 2020) and estimated to balloon into a $61 billion industry by 2034 (Precedence Research, 2025). The market currently offers a variety of solutions spanning from prevention to treatment to provide dietary recommendations, advice, and (food) products based on an individual’s personal, life stage, genetic, metabolic, and lifestyle data (Romero-Tapiador et al., 2023). The premise of personalized nutrition is that individuals have different preferences to reach their health goals (Dijksterhuis et al., 2021) and that individuals respond differently to the same food (Zeevi et al., 2015) with the ultimate goal of improving lifespan and healthspan (Ordovas and Berciano, 2020; Wickramasinghe et al., 2020). Nutritional care, whether for disease prevention or treatment, is recognized as a human right (Cardenas et al., 2023) and should be personalized to the individual. Artificial Intelligence (AI) has been increasingly employed in a number of personalized nutrition solutions, with the aim of simulating human behavior and intelligence through a collection of tools and algorithms, allowing the compiled system to learn and think in ways previously impossible for humans (US GAO, 2022; Detopoulou et al., 2023; Meskó, 2023). The adoption of AI in clinical nutrition in the form of enteral and parenteral nutrition is also gaining traction (Park et al., 2024).

AI can collect, curate, analyze and identify hidden patterns and trends at a speed unmatched by humans. This means that (if trained well), AI can identify correlations between disparate data sets to support healthier choices and increase engagement. In disease prevention, combining bioinformatics and personal data with AI’s learning power could provide experts with deep understanding to create custom nutrition plans and advice, leading to more effective changes in people’s dietary habits. Examples of current market solutions that have integrated AI are outlined in Table 1.

**TABLE 1:** Examples of current applications of AI in Personalized Nutrition.

• Identifying individual biochemical and gut microbiome signatures
• Matching individual preferences and taste to food and ingredient databases
• Creation of 3D printed nutritional supplements
• Predicting blood sugar response to specific meals and interventions
• Predicting disease risk
• Identification of bioactive compounds in food
• Providing real-time feedback via chatbots
• Recognizing and analyzing composition and portion size of foods
• Segmenting users into groups based on dietary patterns or preferences

### The opportunity of AI in nutrition and behaviour change

The personalized nutrition industry faces many challenges new and old, which include: a lack of adherence to recommendations (Davies et al., 2023); limited scientific evidence on the benefit of a personalized approach (Samad et al., 2022); a lack of available practice guidelines (Davies et al., 2023); the high cost associated with developing and training sophisticated AI algorithms; the substantial computational resources required (Vanian and Leswing, 2023); and the lack of trust and adoption of digital health tools by healthcare professionals (US GAO, 2022). AI opens the opportunity to combine disparate data sets, identify barriers to behaviour change and deliver guidance, advice, and support in real-time (Lee, 2023; Hillesheim and Brennan, 2023). To achieve this end goal, the AI system needs to be trained with accurate, representative, and trustworthy data sources. These training datasets require the input of humans who decide what training datasets are included, how they are combined, labelled and how algorithms are developed (Beriain et al., 2022). Herein lies the problem we will discuss next.

#### The ethics of AI and current frameworks

Algorithms are ultimately decisions made by the computer system to provide an output. This can be done without using AI (Zakeri et al., 2022; Wang et al., 2024). However, to handle complex topics such as health and nutrition, AI can be employed to make decisions at a fraction of a second (Sak and Suchodolska, 2021; US GAO, 2022). However, to provide recommendations that are relevant, accessible, and equitable to the individual and benefit wide sections of society, the data used to train algorithms should be free from bias, inclusive and representative (Calvaresi et al., 2022; Detopoulou et al., 2023). Limiting or perpetuating existing inequalities should be avoided at all costs if personalized nutrition is going to benefit all (Renner et al., 2023). Health inequality is already a reality owing to lack of broadband access; limited access to healthcare services due to insurance coverage; and high-cost burden of digital tools, exacerbating the digital divide (Buolamwini, 2019; Zhang, et al., 2023). Therefore, the risk of using biased datasets to train AI systems, will only perpetuate existing biases and lead to widening inequality.

Ethical AI frameworks such as those published by the European Commission (2019), UNESCO (2022), OECD (2019) and AI4People (Floridi, et al., 2018), are useful to ensure the employment of AI that is safe, fair, and beneficial to society. They all emphasize human-centric values through transparency, accountability, safety, and fairness, while highlighting human rights (Fjeld et al., 2020; European Commission, 2019; UNESCO, 2022; OECD, 2019; Floridi et al., 2018). It should be noted that these frameworks apply to AI systems across industries and, while important, lack domain-specificity. Currently, none of the frameworks focuses specifically on nutrition and behaviour change.

The EU framework is most applicable to healthcare (diagnostics, decision support), public services, robotics, consumer tech, and social media, whilst the UNESCO framework mostly applies to education, health, culture, communication, and environment. The AI4People framework focuses on areas such as labor, healthcare, civic engagement, environment, and innovation. All frameworks could be used and applied in nutrition as nutrition intersects both health (accessibility, affordability, social determinants, digital literacy) and food (e.g., production, transparency, accessibility, agriculture).

In addition to the above-mentioned frameworks, adhering to general standards and best practices in AI is also critical to ensure quality and efficacy. Examples include the nearly 300 AI-related standards formulated by various Standards Development Organization, or the vast published and in-progress standards from the International Organization for Standardization (ISO) (AI Standards Hub, 2023; ISO, 2024). In short, in order to shield society from harm, AI systems need to comply with established frameworks and standards.

### Origins of bias in nutrition AI training datasets

#### Unrepresentative data sets

To train AI systems effectively and better predict real-world contexts, solutions need to reflect varied dietary patterns and nutritional needs for smarter personalized recommendations (Renner et al., 2023; Romero-Tapiador et al., 2023; Shandilya et al., 2022; Oh et al., 2021; Stefanidis et al., 2022). However, a recent study found that digital health tools are unequally adopted and distributed amongst society, especially when considering age and ethnicity (Zhang et al., 2023).

AI systems should ensure that dietary recommendations include large portions of the whole population, independent of gender, race, income, education, and health conditions (Atwal, 2024). Despite many new initiatives to limit discrimination and inequality, the risk of using biased datasets is high. In the US, for example, AI algorithms might not represent uninsured patients, older adults, or include non-English languages, leading to skewed algorithm learning (Detopoulou et al., 2023). Additionally, women have historically been excluded from clinical trials until 1992 (Westervelt, 2015; Berlin and Ellenberg, 2009). There is generally a lack of representation of non-Western dietary patterns and foods, inadvertently leading to exclusion and perpetuation of existing bias (Burt, 2021).

#### Lack of contextual data that drives healthy behaviours in a variety of populations

From a behavioural perspective, it is well known that dietary and lifestyle behaviours are key factors to health and longevity (Li et al., 2018; Fang et al., 2021; Santos, 2022). This means that individuals have different preferences, levels of motivation, expectations, levels of health and AI literacy and levels of self-efficacy (Dijksterhuis et al., 2021). Therefore, AI systems need to be built on empathy, and a deep understanding of dietary consumption to retain cultural and social values when identifying patterns, assessing diets, and recommending foods and meals without discriminating or excluding or using persuasion systems (Calvaresi et al., 2022).

#### A black box approach

Developing AI systems in silos that are not transparent, explainable and without expert scrutiny, have been a longstanding criticisms of solutions leveraging AI, known as a black box approach. This approach further contributes to and perpetuates existing biases that risk widening inequality (US GAO, 2022; European Commission, 2019; UNESCO, 2022; Fjeld et al., 2020; Samad et al., 2022; US GAO, 2022). In order to tackle these important issues, a concerted effort from all relevant stakeholders is needed in order to reduce societal risks. We propose a domain-specific framework with a lens on nutrition and behaviour change, to guide the development of ethical and trustworthy AI systems which will be outlined next.

### The 7- pillar framework for the development of ethical and trustworthy AI solutions

The goal of AI in personalized nutrition is to enhance individual health and behavioural outcomes (Detopoulou et al., 2023). To reach that goal, the core focus should be on the ethics of the recommendations provided by AI systems to build trust. Neglecting the ethical, societal, and organizational impact of AI, will lead to social inequity and injustice.

**FIGURE 1:**
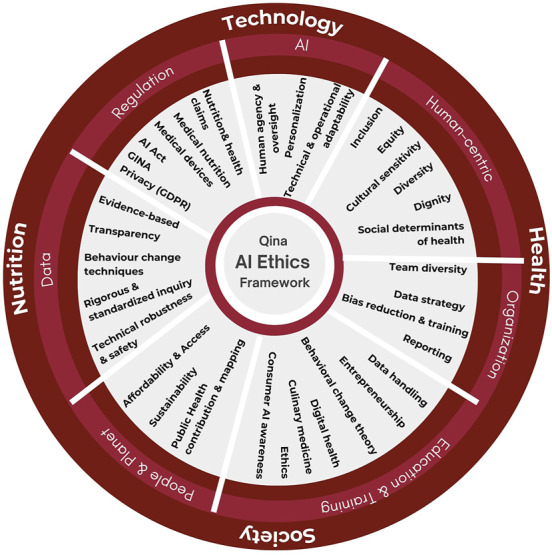
AI ethics framework containing 7 principles: data, AI, human-centricity, people and planet, regulation, organizational commitment, and education and training.

To address the ethical challenges AI poses in nutrition and behaviour change specifically, we propose a comprehensive ethical framework that includes seven interrelated principles which are: data, the AI system, human-centricity, people and planet, regulation, organizational commitment, and education and training (Figure 1).

These principles consider the impact of the AI system on the individual, societal and organizational level, whilst covering the 4 key dimensions of Personalized nutrition which are food, health, technology, and society.

### Data

Data used to train AI systems should be evidence-based, diverse, in line with current nutritional guidelines and the most recent research to provide scientifically sound, inclusive, and personalized dietary recommendations that meet individual health needs. This requires the use of diverse data sets to train the AI system, to avoid irrelevant recommendations to be made for specific individuals or groups (Fatumo et al., 2023). For example, if an AI system uses genetic data from individuals of European or North American ancestry, it may fail to provide appropriate dietary advice for individuals originating from West African countries who are genetically more salt-sensitive and at higher risk for hypertension (Hilliard, 2021).

Behaviour change is an important discipline that largely contributes to the success of health programs (Villinger et al., 2019; Reinders et al., 2023; Barker and Swift, 2009; Calvaresi et al., 2022). At present, the research is limited to the inclusion of a limited range of behavior change techniques (Villinger et al., 2019). It is also not clear what the ideal mix of behaviour change techniques is, and this remains an under-researched area (Reinders et al., 2023). The development of ethical AI systems that influence behaviour, will require transparency in terms of the theoretical model employed, as well as the use of standardized language for behaviour change techniques used to accurately measure outcomes (Calvaresi et al., 2022; Barker and Swift, 2009; Michie et al., 2013).

A Data nutrition label, such as that proposed by the Data Nutrition Project (Data Nutrition Project, 2021) could aid in increasing transparency and standardization of the data used to train AI systems and for conducting research. A data nutrition label that looks like a nutrition facts panel would be included as part of every digital solution that employs AI. This could help consumers in making informed choices on whether the solution matches their goals, beliefs, and demographics. Currently, no such label exists.

### AI system

To ensure high-quality AI-driven personalized nutrition solutions, adherence to evolving AI standards and legislation is crucial to promote technical excellence, data security, and user safety (AI Standards Hub, 2023; ISO, 2024; OECD, 2019). Privacy, safety, and security form the foundation of trust in AI systems (US GAO, 2022), requiring robust measures to safeguard sensitive data, prevent misuse and unauthorized access (UNESCO, 2022; Lekadir et al., 2022; Floridi et al., 2018). Additionally, flexibility is key for tailoring recommendations, incorporating user feedback, adapting to evolving needs and to technological advancements and scientific updates (Oh et al., 2021; Lekadir et al., 2022; Fisher and Rosella, 2022). Adaptability is essential to ensure relevance and accuracy, while also accommodating user growth, safety, and data handling complexity without compromising on performance over time (US GAO, 2022; Stefanidis et al., 2022; Fisher and Rosella, 2022; Manyika, 2022; Renner et al., 2023; Villinger et al., 2019; Zhang et al., 2020).

Therefore, solutions should be user-friendly and enable users to navigate and control their dietary choices with ease and confidence, free from undue pressure and aligned with their personal values, preferences, and goals while guaranteeing safety. The same considerations apply to AI systems when used in the clinical nutrition setting, such as critical care (Kittrell et al., 2024).

### Human-centric

Access to clinical nutrition, healthy food, and relevant information remains a significant challenge for many across the globe (Perrigo, 2023; Lake 2018; Caraballo et al., 2022; Brown et al., 2022). At present, at-risk communities are disproportionately affected by chronic diseases with reasons cited as living in food deserts (Lake, 2018). In fact, 17 million Americans live in food deserts and find it challenging to find healthy food options (Deloitte, 2022). Difficulty in accessing medical care is also commonly cited (Caraballo et al., 2022). Health outcomes are influenced by factors beyond individual choice, such as socioeconomic status and the availability of nutritious food. This widening health inequality is perpetuating the notion that healthy eating is for the wealthy (World Health Organization, 2022). AI solutions, therefore, need to ensure accessibility and equitable treatment to guarantee outcomes that benefit all (Rankin et al., 2018).

This means that integrating social determinants of health (SDOH) into AI solutions is essential for equitable healthcare (Campanera et al., 2023; Minga et al., 2023). For example, by collecting geospatial data from users, AI algorithms can adapt recommendations to better support healthier eating behaviors, and healthier options that are more relevant to prevent risk factors associated with the user’s location (VoPham et al., 2018; Lake, 2018; Rigg, 2022).

Personalization remains at the core of user-friendly AI in nutrition, aiming to tailor nutrition recommendations to an individual’s unique dietary needs and lifestyle choices, improving adherence, experience, and satisfaction (Oh et al., 2021). Users need to have the autonomy to choose freely whether to adopt the nutritional advice or not, considering their unique preferences and needs, along with personal values and goals (Floridi et al., 2018; Zhang et al., 2020; Calvaresi et al., 2022). Continuous feedback should be based on user interactions and biological responses to check on retention and satisfaction, but also adapt to physiological changes, essential for long-term results beyond initial outcomes such as weight loss (Feng et al., 2023). This approach is certainly not new and has been highlighted by previous researchers (Barrocas et al., 2023). While not specifically related to AI, we refer the reader to a comprehensive article on the ethical dimensions nutrition care teams need to consider when dealing with disease-related malnutrition and access to care using the Troubling Trichotomy as the foundation (Barrocas et al., 2023).

### People and planet

Sustainability needs to be built into AI systems to align individual health benefits to environmental sustainability (Davies et al., 2023; Fadnes et al., 2022). This could ultimately extend life expectancy (e.g., Food4HealthyLife calculator) and meet cultural acceptability and affordability. AI can assist in recommending more sustainable diet options and tailor recommendations that support both practitioners and consumers in making sustainable, health-aligned dietary choices, while aligning with personal health goals and individual values (Pettinger et al., 2023). These newly published guidelines could serve as a starting point for developers and data scientists to include sustainability in AI recommendations, ultimately aiding practitioners to provide more actionable advice.

There is also a social responsibility to share learnings and data amongst stakeholders to improve future solutions to meet broader public health goals, effectively addressing global health challenges (Fisher and Rosella, 2022). For instance, AI’s ability to monitor and predict malnutrition across diverse populations paves the way for precise, targeted interventions (Larburu et al., 2022). AI technologies could therefore guide public health policies, aiding in the fight against nutritional deficiencies and promoting sustainable food systems at local, national, and international levels.

### Regulation

Compliance with legal and regulatory standards is vital to ensure that personalized nutrition AI solutions operate within the boundaries of what is legally and ethically recognized (Lekadir et al., 2022; Calvaresi et al., 2022). For example, The AI Act in the EU and the AI Bill of Rights in the US, both recently approved, aim to ensure that AI systems are safe, transparent, traceable, non-discriminatory, and environmentally friendly (European Commission, 2022; The White House, 2022). Other regulations that could apply in Personalized nutrition, could span beyond GDPR (May 2018) and include medical nutrition, EFSA, HIPAA, Medical devices regulation (MDR), and GINA. Therefore, companies operating or developing solutions should seek legal counsel to ensure they comply.

### Organizational commitment

The lack of diversity within the technology industry, both in skill sets and backgrounds, impacts the quality and diversity of the AI solutions developed. The introduction of unconscious biases in the development of AI systems poses a societal risk (World Economic Forum, 2021; Buolamwini, 2019; Zinzuwadia and Singh, 2022; US GAO, 2022). This problem is exacerbated by the underrepresentation of women and in particular, people of color in technology (World Economic Forum, 2021; Buolamwini, 2019; BCS - The Chartered Institute for IT, 2022). To illustrate, women account for less than 25% of AI specialists globally (World Economic Forum, 2021). Furthermore, recent studies have spotlighted AI’s failures in correctly identifying the gender of darker-skinned women compared to their lighter-skinned counterparts (Wetsman, 2022; Zinzuwadia and Singh, 2022), a technology increasingly used in consumer nutrition and health solutions. This racial and gender bias is partly attributed to the lack of diversity in the data science community and health technology sector in general, which if not repaired, could worsen existing inequities (Buolamwini, 2019).

For consumers and healthcare practitioners to trust both the industry solutions, and the skills of the employees developing AI systems, organizations will need to be more transparent on who is behind any AI solution. For example, companies can make sure that their team is visible (on the website) and adequately trained in areas other than their domain expertise (Poínhos et al., 2017). Organizations can also be more transparent about their policies and training systems in place to address bias, social impact, and potential harmful concerns. Transparency is key for building trust.

### Education

In the current healthcare landscape, there is a readiness gap among professionals regarding the adoption of AI technologies (Abrahams et al., 2019). This derives mainly from a lack of understanding of the tangible benefits that AI can offer, and the perceived threat it poses to traditional roles within the healthcare system (Sak and Suchodolska, 2021; Abrahams et al., 2019). Concerns about the “dehumanization” of care have been raised despite the recognition of AI’s potential to augment healthcare services (Detopoulou et al., 2023). There is a need to invest in training internally and externally, to ensure that employers, partners, and users (both healthcare professionals and patients/consumers) have the skills to leverage AI ethically and confidently. For AI in personalized nutrition to realize its full potential, education, and training to healthcare professionals must evolve to include: behavioural design thinking, creative problem-solving, data handling, entrepreneurship, behaviour change theories, ethics in AI, and culinary medicine.

## Conclusion

As we stand at the crossroads of technological innovation and ethical responsibility, the personalized nutrition sector is uniquely positioned to leverage AI to impact individual health and wellness on a large scale. The 7 principles of ethical and trustworthy AI at the intersection of nutrition and behavior change presented here provide a compass to navigate the rapidly unfolding AI landscape, ensuring that advancements are not only scientifically and technologically sound but also morally grounded. To date, the personalized nutrition sector has made significant strides in developing solutions that cater to an individual’s personal and biological data, yet a lot more work and research is needed to ensure the benefits are reaped equitably through multidisciplinary and inter-industry collaborations in order to improve health.

## Data Availability

The original contributions presented in the study are included in the article/supplementary material, further inquiries can be directed to the corresponding author.
